# Supplementary material for: Chloroplast genomes of Rubiaceae: Comparative genomics and molecular phylogeny in subfamily Ixoroideae
Source: PLoS One. 2020 Apr 30;15(4):e0232295. doi: 10.1371/journal.pone.0232295 (PMC7192488; doi:10.1371/journal.pone.0232295)
Supplement: Attachment — Submitted filename: Responses-to-reviewers.docx [file pone.0232295.s006.docx]

**PONE-D-19-35274 Responses to Reviewers.**

**Thanks to reviewers for their valuable suggestions. Please find below our answers.**

**Reviewer #1**: “The research article is overall good, but many minor errors need to be removed prior to publishing. Tables including supplementary tables contains may space and other spelling, scientific nomenclature errors and the tables format. Therefore, my suggestion is to revise the manuscript thoroughly”.

Ans: The manuscript has been revised thoroughly.

“Abstract should be rewritten according to the PLos ONE”.

Ans: Done: Describe the main objective(s) of the study -> OK ; Explain how the study was done, including any model organisms used, without methodological detail -> Ok ; Summarize the most important results and their significance -> Ok ; Not exceed 300 words -> OK ; Abstracts should not include Citations -> Ok

“Many spelling errors should be revised, here only give some examples: Line 3 of introduction: ‘......13.600 species......’ should be ‘.......13,600 species.......’? ‘......such as Vangueria, Alibertia and Duroia L.f.’: scientific nomenclature should be consistent.  ”

Ans: Done. The changes have been highlighter along the manuscript.

**Reviewer #2:** “the authors provided whole cp genome sequences for 27 species of the Rubiaceae subfamily Ixoroideae which sequeced using high-throughput genome sequencing method, analyzed the plastid genome structure and the chloroplast genome evolution in the Rubiaceae through efficient methodology for de novo assembly of plastid genomes. this work is meaningful for the molecular phylogeny analysis in Rubiaceae subfamily Ixoroideae.”

Some suggestion:

“1) What is the role played by the second main objectives of this paper, where "to test the efficiency of mining SNPs in the nuclear genome of Ixoroideae based on the use of a coffee reference genome to produce well-supported nuclear trees"，support the chloroplast genome results?”

Ans: Mining SNP using the C. canephora genome has been tested (Hamon et al., 2017) and was successful. An average of 79% of the SNP determined in the Coffea genus are transferable to Ixoroideae, with variation ranging from 35% to 96%. A sentence has been introduced in the Abstract and a chapter called “**Nuclear SNP mining and Efficiency of transferability of methods from Coffea to Ixoroideae” in Results and Discussion**“ has been reorganized to explain better this part.

“2) The content of the discussion part and the result part are repeated, so it is suggested that merged these two parts.”

Ans: Done. We reorganize the manuscript with a Results and Discussion chapter.
